# Amplification biases: possible differences among deviating gene expressions

**DOI:** 10.1186/1471-2164-9-46

**Published:** 2008-01-28

**Authors:** Séverine A Degrelle, Christelle Hennequet-Antier, Hélène Chiapello, Karine Piot-Kaminski, Francois Piumi, Stéphane Robin, Jean-Paul Renard, Isabelle Hue

**Affiliations:** 1 Biologie du Développement et Reproduction UMR 1198; ENVA; CNRS, FRE 2857, Institut National de la Recherche Agronomique, F-78350 Jouy-en-Josas, France; 2 Mathématique, Informatique et Génome UR1077, Institut National de la Recherche Agronomique, F-78350 Jouy-en-Josas, France; 3 Radiobiologie et Etude du Génome UMR INRA/CEA, Institut National de la Recherche Agronomique, F-78350 Jouy-en-Josas, France; 4 Mathématiques et Informatique Appliquées UMR INAPG/ENGREF/INRA 518, F-75005 Paris, France; 5 Station de Recherches Avicoles, Institut National de la Recherche Agronomique, F-37380 Nouzilly, France; 6 Modélisation et Ingénierie des Systèmes Complexes pour le Diagnostic FRE3009 CNRS/BIO-RAD, F-34184 Montpellier Cedex 4, France; 7 Biologie des Champignons Filamenteux UFR ESIL, F-13288Marseille Cedex 09, France

## Abstract

**Background:**

Gene expression profiling has become a tool of choice to study pathological or developmental questions but in most cases the material is scarce and requires sample amplification. Two main procedures have been used: in vitro transcription (IVT) and polymerase chain reaction (PCR), the former known as linear and the latter as exponential. Previous reports identified enzymatic pitfalls in PCR and IVT protocols; however the possible differences between the sequences affected by these amplification defaults were only rarely explored.

**Results:**

Screening a bovine cDNA array dedicated to embryonic stages with embryonic (n = 3) and somatic tissues (n = 2), we proceeded to moderate amplifications starting from 1 μg of total RNA (global PCR or IVT one round). Whatever the tissue, 16% of the probes were involved in deviating gene expressions due to amplification defaults. These distortions were likely due to the molecular features of the affected sequences (position within a gene, GC content, hairpin number) but also to the relative abundance of these transcripts within the tissues. These deviating genes mainly encoded housekeeping genes from physiological or cellular processes (70%) and constituted 2 subsets which did not overlap (molecular features, signal intensities, gene ID). However, the differential expressions identified between embryonic stages were both reliable (minor intersect with biased expressions) and relevant (biologically validated). In addition, the relative expression levels of those genes were biologically similar between amplified and unamplified samples.

**Conclusion:**

Conversely to the most recent reports which challenged the use of intense amplification procedures on minute amounts of RNA, we chose moderate PCR and IVT amplifications for our gene profiling study. Conclusively, it appeared that systematic biases arose even with moderate amplification procedures, independently of (i) the sample used: brain, ovary or embryos, (ii) the enzymatic properties initially inferred (exponential or linear) and (iii) the preliminary optimization of the protocols. Moreover the use of an in-house developed array, small-sized but well suited to the tissues we worked with, was of real interest for the search of differential expressions.

## Background

Several years ago gene expression profiling has emerged as a tool of choice to study developmental kinetics [1] and is now widely used to study mammalian oocytes or embryos (mouse: [2]; bovine: [3,4], human [5], porcine [6]) including questions on cell lineage differences [7]. However intermingled cells within complex tissues, biopsies, early embryos or single cells give rise to ng or pg amounts of RNA so that amplification has become a prerequisite, coupled sometimes to laser capture micro-dissection (for example [8]).

Two amplification methods have been reported in the 90ties [9,10], a linear procedure based on the use of In Vitro Transcription (IVT) and an exponential procedure based on the use of the Polymerase Chain Reaction (PCR). These exponential and linear definitions have been based on the dynamics of the corresponding enzymatic reactions with no implicit reference to their intrinsic pitfalls. However, it has quickly become "obvious" that the linear process was a high-fidelity process which guaranteed the conservation of the initial transcript abundances [11]. It thus became the tool of choice for gene profiling studies on cDNA and oligo-nucleotide arrays [12]. In the meantime, global PCR amplification procedures have been optimised and claimed better than IVT for array screening when starting from the sub-pg quantities of RNA isolated from single cells [7,13]. As a consequence, numerous reports have compared the performance of PCR and IVT on decreasing amounts of starting material. Some of these extended the comparison to mRNA or total RNA as un-amplified standards although these standards appeared debatable [14].

Since their first use, both linear and exponential amplification processes became commercially available (reviewed in [14,15]) and evolved into 8 to 10 different protocols. The original IVT protocol [16] has been worked out by Baugh et al. [17] to make it more specific, by Moll et al. [18] to make it more efficient and by Schlingemann et al. [19] to adapt it to oligo-arrays composed of sense oriented oligonucleotides. Similarly, the original PCR protocol has evolved into a new amplification process known as the SMART protocol [20]. Several reports compared SMART amplified targets to IVT [21] and/or global PCR [22]. Improvements of the original Brady's protocol have also been worked out and compared to total RNA [7]. At last, alternatives to IVT or PCR have also been proposed such as a PCR step followed by an IVT step, an IVT step followed by a PCR step, the use of single-stranded cDNA instead of double-stranded cDNA (ribo-SPIA protocol) or the use of subtraction prior to RNA amplification (STAR protocol). These recent procedures have also been compared to IVT or total RNA and sound promising (reviewed in [14,15]). They fall beyond the scope of this paper.

In all these studies fidelity, reproducibility and linearity of RNA amplification has been a major concern and increasingly refined statistics have been used accordingly (correlation, fold change, T-test, ANOVA; [23]) to identify amplification biases through deviating expression patterns between amplified and un-amplified material. However, the possible differences between the sequences affected by PCR and IVT amplification defaults were only rarely explored. We thus aimed at studying the biases of moderate amplification protocols as well as their major characteristics, using an in-house developed protocol for the global PCR amplification [24,25] and taking into account in-house criteria linked to our biological purposes. We thus decided to use a small set of in vivo elongating embryos, recovered after uterus flushing, to screen different arrays with the same embryonic material (this study, [26,27]). Second, we chose a bovine array dedicated to these bovine stages rather than a larger Affymetrix array where sequences from this embryonic repertoire were not present. Third, we preferred moderate amplifications to intense ones, as routinely practiced on oocytes and earlier embryonic stages, since intense amplifications cost more and have drawbacks too.

On this basis, we showed that deviating expressions affected 16% of the array after PCR or IVT amplifications, formed 2 gene subsets which did not overlap (molecular features, signal intensities, gene ID) and corresponded to housekeeping genes from physiological or cellular processes. Nevertheless, differential expressions were relevant and displayed relative expression levels which were biologically similar, though not identical, between amplified and unamplified samples.

## Results

### Experimental design

Our purpose was to analyse moderate amplifications on tissues of similar but also different molecular complexities to analyse the relevance and biases in gene profiling studies following RNA amplification. To this aim, we selected two protocols (IVT one round and global PCR) and five tissues: three embryonic tissues of equivalent molecular complexities as revealed by SAGE data on ovoid, tubular and filamentous stages in pig [28] and two adult tissues of different complexities (brain and ovary) as described by human SAGE and EST data [29].

**Figure 1:**
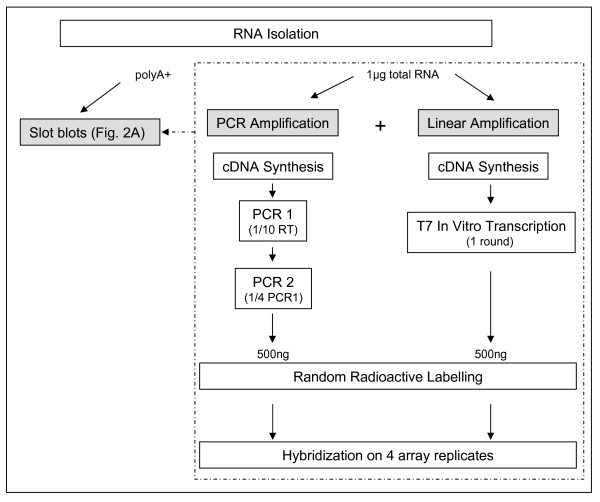
**Experimental design**. Description of the technical steps involved in the experiments designed to analyse the relevance and biases occurring in our gene profiling studies following moderate RNA amplification: IVT one round or global PCR (details on the protocol in the Methods). In this design technical replicates included both target replicates (independently amplified targets) and array replicates (identical copies of the array).

To focus the design on technological variability, we reduced the biological variability as much as we could (Fig. 1). For example, the tissues (embryos, brain and ovaries) were collected on a limited number of pregnant cows. Total RNA from brain and ovary was extracted from different pieces of tissue and pooled thereafter to get a single RNA pool for each tissue. Similarly, RNA from individual embryos was extracted and pooled per stage since biological pooling was acceptable there due to our methodological focus [30]. Global PCR or IVT one round was then applied to 1 μg of total RNA from each tissue. Concomitantly, poly A+ RNA from brain and ovary was purified from the corresponding pools of total RNA and used in slot-blots to assess the biological quality of the amplified material. This has not been done on embryos due to a limited amount of material. We chose slot-blots instead of real-time PCR to assess the quality of the amplified material since a validation of a global PCR by a PCR did not seem reasonable to us due to similar enzymatic drawbacks.

Amplified material from each tissue (brain, ovary, ovoid embryos, tubular embryos and filamentous embryos) was indirectly labelled using "random" hexamers. As advised [31] replicates were emphasized and deliberately focused on technical points. Three or two independent targets for each tissue and each protocol (target replicates) were thus generated and hybridised to 4 replicates of the same array (array replicates), so that 48 measurements per probe were generated for somatic (3 targets × 2 tissues × 2 protocols × 4 arrays) and embryonic samples (2 targets × 3 tissues × 2 protocols × 4 arrays). To find out gene expression differences between protocols or embryonic stages, appropriate statistical analyses have been applied on each set of data.

### RNA amplifications: optimisation and quality

Since drawbacks were reported for both IVT and PCR based protocols which could originate from a too long IVT (degradation effect reported by Spiess [32]) or too many cycles of PCR (saturation effect reported by Cha or Nagy [33,34]), we first challenged our protocols on our tissues to define optimal amplification conditions. The protocols were tested using increasing in vitro transcription time or PCR cycle number, with a special look at 5 transcripts: 3 endogenous and 1 exogenous transcripts in brain and ovary, 1 endogenous transcript in developing embryos. Transcripts encoding EF1α, L23a and Cytochrome oxidase III were selected as somatic controls because of a differential expression between brain and ovary, an easy detection on slot blots with poly A+ RNA and a different length: 1.7, 0.9 and 0.7 kb, respectively (preliminary data, not shown). As a result, an in vitro transcription of 10 h and 2 rounds of 12 PCR cycles on 1/10 of the reverse transcription looked optimal since (i) the size of the "spiking" transcripts was conserved (ii) no shortage or degradation of the amplified material was observed and (iii) the amount of spiking transcripts had increased linearly with the time of the IVT or the number of cycles in the PCR [see Additional file 1].

Comparing the 3 amplified targets generated on the somatic tissues, it appeared that the anti-sense RNA (or aRNA) obtained after IVT corresponded to molecules of 0.1 to 4 kb with a mean size of 600 bp while the cDNA fragments generated by global PCR were reduced in size: 0.1 to 1 kb, with a mean size of 150 bp [see Additional file 2A–B]. Interestingly cDNA populations were similar for brain and ovary (panel B) whereas aRNA populations displayed slightly different patterns (panel A). However, the 3 aRNA targets generated from brain or ovary were similar [see Additional file 2C]. These results underlined a good reproducibility in the production of target replicates, a slightly different distribution of RNA species between tissues with IVT and a more homogeneous pattern between tissues with PCR.

**Figure 2:**
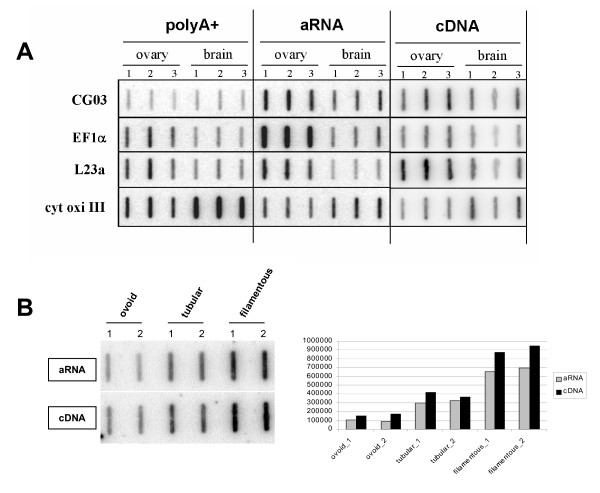
**Quality of the somatic and embryonic amplified products**. 125 ng of each amplified material (aRNA or cDNA) has been spotted onto a membrane and hybridised with DNA probes encoding exogenous (CG03) or endogenous controls (somatic: EF1α, L23a, cytochrome oxidase III; embryonic: IFN-tau). Each replicate (1 to 3) has been generated independently with each protocol (IVT or PCR) on each tissue (A: brain, ovary; B: ovoid, tubular and filamentous bovine embryos). PolyA+ RNA was used as standard for somatic tissues only (A).

To further assess the quality of the amplified material generated on each tissue by IVT or PCR, we took advantage of the endogenous and exogenous transcripts which were used to calibrate the protocols and studied their expression in amplified versus un-amplified material (somatic samples: Fig. 2A, embryonic samples, Fig. 2B). The L23a mRNA, slightly more expressed in ovary than in brain according to poly A+ RNA, kept the same profile after amplification (IVT or PCR). Conversely, the stronger expression of EF1α in the ovary was either increased (IVT) or attenuated (PCR) whereas the stronger expression in brain of the Cytochrome Oxidase III mRNA was weakened after IVT and abolished after PCR. Interestingly enough the exogenous mRNA, which was equally added in the RNA from brain and ovary (see Methods), appeared more expressed in the ovary after IVT but not after PCR. Using the Interferon-tau transcript as endogenous control for bovine developing embryos (Fig. 2B) we showed that the amount of this sequence increased from the ovoid to the filamentous stage after amplification (IVT or PCR) as it does *in vivo *(reviewed in [35]). On this very small set of transcripts it was obvious that under and over representations occurred during amplification, but to which extent and with which impact on gene expression differences?

### Global features of somatic and embryonic hybridisations

As previously advised by N'Guyen [36], we first determined the amount of labelled target to be used for each hybridisation so that no additional signal appeared but the intensity of the positive signals increased when the amount of target did (50, 125, 250 and 500 ng for aRNA or cDNA labelled targets; data not shown). On this basis, 125 ng of each target has been hybridised to each array with no particular focus on the relative amplification rates and the subsequent equivalence between these targets.

**Figure 3:**
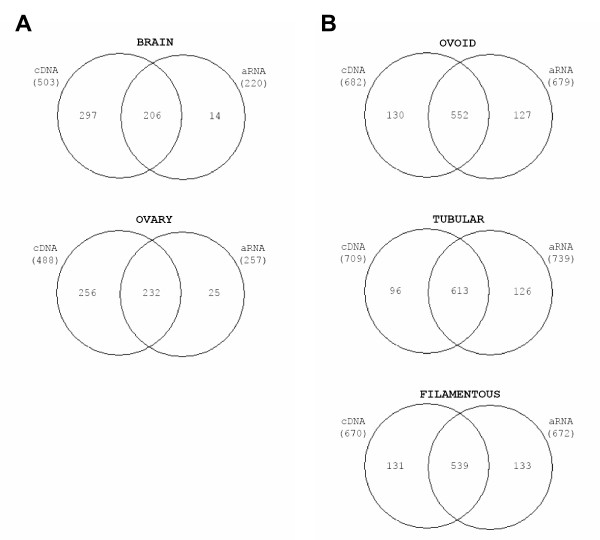
**Venn diagrams on somatic (A) and embryonic (B) hybridisations**. Only valid signals were displayed here after hybridisation on the array. A signal was considered "valid" when the Imagene software did not flag it (flag = 0) and when the same signal was observed on 2 thirds of the arrays, namely: 8 out of 12 for the somatic ones and 5 out of 8 for the embryonic ones.

Considering the somatic hybridisations, PCR amplified targets gave a double amount of valid signals as compared to IVT amplified ones (Fig. 3A), when valid meant observed on 2 thirds of the arrays. In this case, common signals represented 90% of the IVT signals but only 45% of the PCR ones. This however was not true when valid meant detected on all the arrays with intensities 2 times over the background. Indeed, such a stringent calculation gave similar numbers of signals with IVT or PCR amplifications: 112 versus 96 with brain and 167 versus 146 with ovary, respectively. Conversely, embryonic targets (Fig. 3B) displayed similar numbers of signals at each stage but 2 to 3 times more signals than somatic ones, as expected from an array enriched in embryonic probes. Moreover, common signals between embryonic targets represented 70 to 80% of the signals generated by IVT and PCR amplifications.

**Figure 4:**
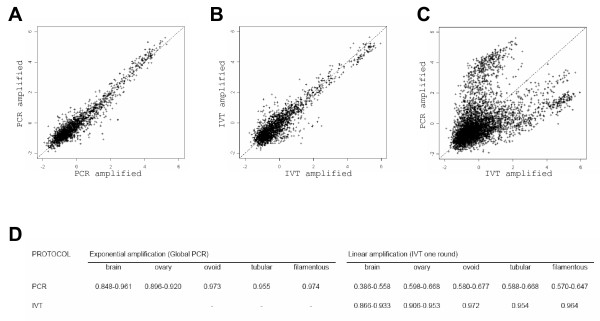
**Scatter plots between hybridisations with bovine adult brain (A-C) and correlations within the whole design (D)**. Signal mean intensities were calculated for each amplified target on 4 arrays and plotted pair-wise per replicate. For example, PCR-amplified: replicate 1 versus replicate 2 (A) and IVT-amplified: replicate 1 versus replicate 2 (B). Similar results were obtained for the 4 other pairs (1 versus 3 and 2 versus 3). Signal mean intensities per protocol were calculated on 12 arrays (4 arrays per replicate × 3 replicates) and plotted pair-wise per protocol (C): PCR-amplified versus IVT-amplified. Similar results were obtained for the ovary and the embryos (ovoid, tubular and filamentous stages). Correlation factors between hybridisation profiles (D).

When analysed per protocol, the technical replicates (target replicates: 3 for the somatic tissues; 2 for the embryonic stages; array replicates: 4 per target) proved to be nicely correlated as evidenced by the corresponding scatter plots (Fig. 4A–B). Briefly, the coefficients of correlation were between 0.85–0.97 for the hybridisations after PCR or IVT amplification: 0.85 to 0.96 for the somatic signals and 0.95 to 0.97 for the embryonic ones (Fig. 4D). However, the correlation between PCR and IVT amplified products was much lower (0.39 to 0.67 for somatic hybridisations; 0.58 to 0.67 for embryonic ones; Fig. 4C–D). The scatter plots revealed additionally a large number of signals with a very low coefficient of correlation which corresponded to signals of high intensity with PCR amplifications but low intensity with IVT and *vice versa *(Fig. 4C). These signals generated a crab-like figure of high interest with respect to amplification distortions.

We thus confirmed a high correlation within methods, an intermediate correlation between methods and evidenced a divergence between methods for at least a subset of the array. We thus aimed at its characterisation.

### Amplification distortions in somatic and embryonic hybridisations

**Figure 5:**
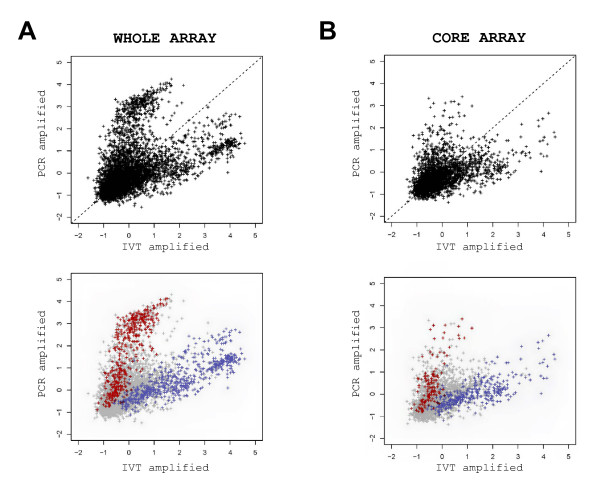
**Evidence for gene expression differences between PCR and IVT amplified samples**. Signal mean intensities per protocol were calculated on 24 arrays (4 arrays per target × 3 targets × 1 tissue = brain) and plotted pair-wise per protocol: PCR-amplified versus IVT-amplified using either the whole set of data (A) or its biological core (B). Coloured, the signals identified as significant gene expression differences between protocols whatever the tissues (n = 154; red: global PCR; blue: IVT one round).

Gene expression differences between amplification methods (global PCR and IVT one round) were identified with the TMEV 3.0 software. Analysing the whole array (1920 EST), 341 gene expression differences were identified whatever the tissues. Interestingly most of them were localised into the "crab claws" previously identified in the scatter plots between PCR and IVT amplified samples (Fig. 5A). Before any other analysis this result suggested that these differences were amplification discrepancies. Repeating the analysis on the core array (987 EST, see Methods), 154 gene expression differences appeared significant between PCR and IVT amplified samples. These ones localised differently (Fig. 5B), showing that the crab claws were mostly due to the mitochondrial sequences (among which 12SrRNA) which were largely redundant (33%) within the whole array. Though biologically not challenging, this drawback (due to the fact that the embryonic library was neither normalised nor sequenced when arrayed) highlighted a specific PCR bias towards 12SrRNA sequences which could not be visualised on arrays containing highly selected cDNAs. Reverse transcription being mostly achieved on total RNA, this however was of methodological interest since those sequences incorporate a part of the isotope during the labelling.

**Figure 6:**
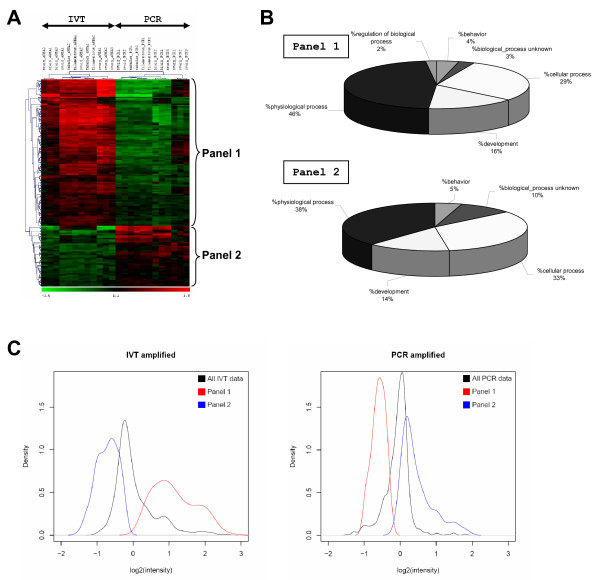
**Characterisation of PCR and IVT amplification biases**. Unsupervised hierarchical clustering of the significant gene expression differences (n = 154) identified between PCR and IVT amplified samples whatever the tissues (A). Biological processes concerned by these gene expression distortions as defined by a search through Gene Ontologies (B). Representations of the deviating gene expressions on the core array: n = 987 EST as compared to the whole IVT and PCR datasets (C): the black lines correspond to the distribution of the intensities in each dataset; the red lines and the blue lines correspond respectively to densities of the deviating expressions from Panel 1 and 2.

Applying a clustering analysis to the relevant differences identified between amplified samples from embryonic and somatic tissues (n = 154), 109 appeared at first glance attributable to IVT and 45 to PCR (Fig. 6A). However, in the absence of unamplified standards such as mRNA targets or total RNA targets, one cannot distinguish higher IVT expressions due to IVT induced over-expressions or PCR induced under-expressions, and *vice versa*. We thus named these groups of genes Panel 1 and Panel 2 instead of IVT and PCR. Most of these deviating expressions corresponded to genes involved in similar processes: 75% and 71% in physiological and cellular processes, respectively (Fig. 6B). As expected from the clustering results, the deviating gene differences from Panel 1 fell into the highest intensities of the IVT data and lowest intensities of the PCR data whereas those from Panel 2 showed the opposite distribution (Fig. 6C). Obviously, the density of these deviating genes over unamplified data would have been of high interest to sort out the part of IVT and/or PCR defaults in these deviating expression differences. 64 and 32 genes referenced in the Unigene Bos taurus index were respectively identified within Panel 1 and Panel 2 [see Additional files 3 and 4]. In Panel 1, the endogenous controls encoding RPL23a and EF1α were recognised thus confirming that some of the deviating expressions we observed on Fig. 6 were due to IVT induced over-expressions (see Fig. 2). To further know whether molecular features such as transcript size, GC content or presence of hairpins could partly explain such deviations, we explored these features on the amplification affected sequences from both Panels.

**Figure 7:**
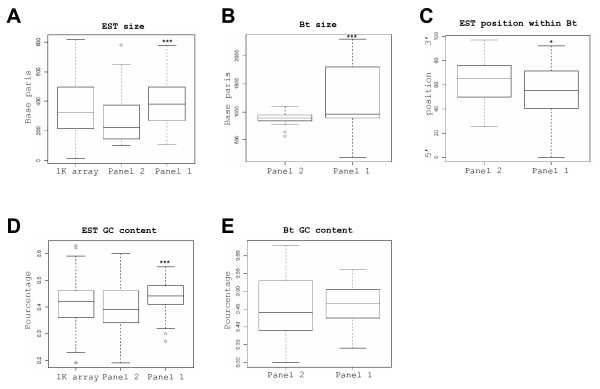
**Molecular features of the gene subsets affected by amplification defaults**. The EST from Pane1 1 (n = 109) and Panel 2 (n = 45) were compared to the EST of the core array (or 1 K array; n = 987) whenever interesting. The distributions of EST size, Bt. size and EST positions within the referenced transcripts from the Bos taurus Unigene index (or Bt.) were represented by box plots (A-C). Sizes were expressed in base pairs. Positions within the Bt. were expressed as % of the whole Bt. size, starting from the 5' end which is defined here as 0. The GC content in these subsets and in the corresponding Bt. was also represented by box plots (D-E). Boxes from the box-plots extended from the 25^th ^percentile to the 75^th ^percentile with a horizontal bar representing the median. Statistical significance between median EST size, Bt. size, EST positions and GC contents has been estimated with T tests (null hypothesis: no differences). (*) means significant (P < 0.05) and (***) highly significant (P < 0.01).

We first found (Fig. 7A–E) that the sequences from Panel 2 (i) displayed a reduced size as compared to those from Panel 1: 200 pb against 350 pb (ii) corresponded to significantly smaller sized cDNAs: 850–950 pb against 900–1800 pb (iii) were more frequently located in the 3' end of the cDNAs: 25 to 50% of the Bt. length and (iv) displayed a lower GC content. Nevertheless, this last difference stopped being significant when the full length cDNAs were compared (Fig. 7D–E) likely due to a "buffering" effect of the coding regions where the GC contents are often closer to 45%. Considering hairpins as potential pausing sites, dA stretches as internal oligo-dT priming sites and promoter-like sequences as alternative RNA polymerase initiating sites, we then observed that sequences from Panel 2 contained also more hairpins (60% versus 37%) and A stretches (10% versus 5%) than those from Panel 1. They displayed however similar contents of promoter-like sequences (Table 1).

**Table 1:** Additional features on the gene subsets affected by amplification defaults. Hairpins, A stretches and promoter like sequences have been investigated. The parameters were the following: hairpins (minimal length: 10 nucleotides, maximal length: 100, maximal gap: 50), A stretches (size: 18A, maximal gap: 3), promoter of the T7 RNA polymerase (forward sequence: CCCTATAGTGAGTCGTATTA and reverse sequence, maximal gap: 6). Results on Panel 1 and 2 are summarised here. Statistical significance between subsets or features has not been evaluated.

	**panel 1**	**panel 2**
palindromes		
≥1	24/30 (80%)	45/65 (75%)
≥ 2	18/30 (60%)	22/60 (37%)
>2	9/30 (30%)	10/60 (17%)
dA streches	3/30 (10%)	3/60 (5%)
promoter-like	8/30 (27%)	12/60 (20%)

Conclusively, it appeared that systematic biases arose during both amplification procedures independently of (i) the sample used: brain, ovary or embryos, (ii) the enzymatic properties initially inferred (exponential or linear) and (iii) the preliminary optimisation of the protocols. These distortions affected 16% of the core array (154/987) and involved different subsets of genes (Panels 1 and 2) which harboured different molecular properties.

### Gene expression differences between embryonic stages

Knowing from above that systematic biases arose during amplification (global PCR and IVT one round) and affected 16% of the core array (987 EST), we wondered whether gene expression differences identified between embryonic stages with amplified samples could be both reliable and relevant.

**Figure 8:**
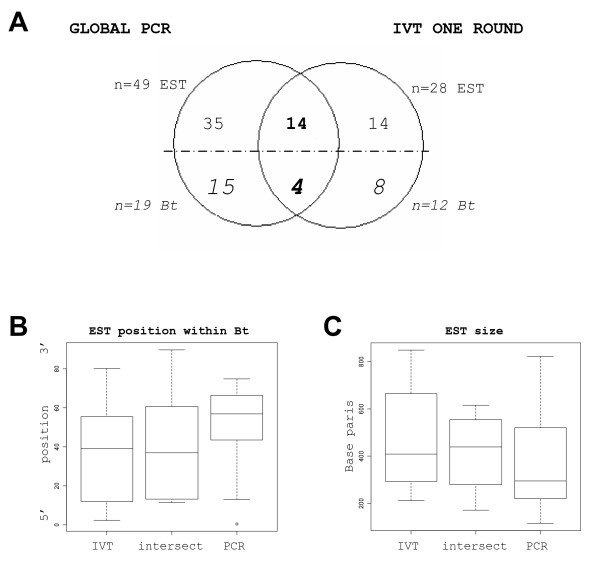
**Differential expressions between embryonic stages**. Venn diagrams on gene expression differences identified between embryonic stages with PCR and IVT amplified samples (A). The distribution of the EST positions within the referenced transcripts from the Unigene Bos taurus index (or Bt.) and the distribution of the EST size were represented by box plots (B, C). Positions within the Bt. were expressed as % of the whole Bt. size, starting from the 5' end which is defined here as 0 (B). Sizes were expressed in base pairs (C). Boxes from the box-plots extended from the 25^th ^percentile to the 75^th ^percentile with a horizontal bar representing the median. Statistical significance between median EST size or EST positions has not been evaluated.

49 gene expression differences were identified between stages (ovoid, tubular and filamentous) with PCR amplified samples and 28 with IVT amplified ones. Among these, 14 were IVT specific, 35 PCR specific and 14 were commonly identified (Fig. 8A). The common ones (n = 14 EST) encoded 4 genes referenced in the Unigene Bos taurus index and corresponded to transcripts identified in another study using IVT amplified samples only [26]. We showed therein that c12, c93, c88 and TKDP1 transcripts were differentially expressed among these stages (c12, c93: Northern blots; TKDP1 [37]). The IVT specific differences (n = 14) encoded 8 genes referenced in the Bos taurus index, 4 of which were known as reliable differential expressions: IFN-tau (our endogenous control for embryos, Fig. 2B), Cox2 [38], c12 and PAG11 [26]. Similarly, the PCR specific differences (n = 35) encoded 15 genes referenced in the Bos taurus index, 5 of which were also known as reliable differences: c12, c93, TKDP1, PAG11 and IFN-tau. Surprisingly, they were not identified as common differences between PCR and IVT amplified samples. Looking in more details at the corresponding EST it appeared clearly that, although located in the same Bt., they did not overlap. Extending this analysis to the list of specific differences (n = 49; 35 +14) we found that the EST from the PCR group were frequently located at the 3'end of the referenced cDNAs (or Bt.), as compared to those from the IVT group (Fig. 8B), and displayed reduced sizes (Fig. 8C). Last but not least, a few differences identified between embryonic stages with PCR amplified samples (2 Bt./15) matched with those identified in Panel 2 (2 Bt./32) whereas no intercept was detected with Panel 1.

Since these differential patterns were detected with amplified embryonic targets, we compared their relative expression ratios between amplified and unamplified RNA (Table 2). We thus quantified c12, c93 and PAG11 expression levels between stages as revealed by former Northern blots [26] and performed Real-Time PCR on 2 new transcripts: Cox2 (identified only by IVT targets) and IFN-tau (identified by IVT and PCR targets through non overlapping EST). From these results, one clearly sees that the differential ratios between stages were biologically similar, though not identical. Indeed, the differential ratios for Cox2 looked smaller at some stages with IVT targets whereas the differential ratios of IFN-tau, c12 and PAG11 appeared smaller with PCR targets. Nevertheless, only one inverted ratio appeared between IVT and PCR: the c12 ratio between tubular and filamentous stages.

**Table 2:** Comparison of the differential expression ratios observed between embryonic stages using amplified and unamplified material. The expression ratios observed with amplified targets (IVT and PCR) come from the array datasets presented in this study. Those originating from Real-Time PCR come from the gene specific validations (IFN-tau, Cox2) we performed on unamplified RNA from each stage. Those originating from Northern blots (c12, c93, PAG11) come from previous results on unamplified RNA from each stage [26].

	**Array**						**Northern**			**Real-time PCR**		
	**IVT amplified material**			**PCR amplified material**			**(mRNA/18S)**			**(mRNA/Gapdh)**		
	
**Gene**	**ov/tub**	**tub/fil**	**ov/fil**	**ov/tub**	**tub/fil**	**ov/fil**	**ov/tub**	**tub/fil**	**ov/fil**	**ov/tub**	**tub/fil**	**ov/fil**
**IFN-tau**	-3,21	-1,57	-6,94	-2,4	-2,31	-5,55				-5,48	-1,57	-8,63
**Cox2**	-1,72	-1,26	-2,17	-1,22	-1,73	-2,10				-4,32	-1,74	-7,53
**c12**	4,93	3,86	8,51	1,87	**-1,26**	1,48	3,24	2,63	4,02			
**c93**	-1,2	-1,83	-2,19	-1,66	-1,62	-2,67	-1,52	-2,87	-4,37			
**PAG11**	-1,52	-2,87	-4,37	-1,49	-1,06	-1,58	-2,98	-1,3	-3,87			

As a final view, gene expression differences identified between embryonic stages with amplified samples were both reliable (tiny intersect with deviating expressions) and relevant (biologically valid). In addition, the molecular features observed on the differential EST identified by IVT or PCR amplifications suggest that global PCR favoured the representation of short cDNA harbouring rather low GC contents.

## Discussion

This work illustrated the questions frequently asked since 2002 about RNA amplification and showed that even with optimised and reproducible protocols deviating gene expressions affected 16% of our array and appeared whatever the tissue. These biases, linked to the abundance or the molecular features of the sequences affected by amplification defaults, corresponded mainly to housekeeping genes from physiological and cellular processes. Differential expressions, however, were found reliable and relevant with biologically similar expression ratios between amplified and unamplified material.

Similar biases were reported in previous studies using also moderate IVT and PCR amplifications. They evidenced either contradictory expression ratios or missing spots [39,40] but also a vast majority of expression patterns which differed only in the magnitude of the differential expression [41]. In our study, only one gene out of the five tested showed an inversed ratio at one stage after PCR amplification, whereas most of them showed ratios which differed only in their magnitudes. All of them however were relevant as confirmed by Northern blots or Real-Time PCR. Interestingly, the deviating genes from our study corresponded mainly to housekeeping genes whereas those identified by van Haaften (genes lost during IVT amplification) rather included transcription factors. As an alternative to minimise distortions, Real-Time detection of amplified products has been proposed to prevent over-amplification in PCR-based protocols [34] and a similar approach has been used before and after IVT amplifications to discriminate between well and badly amplified samples [40]. This has also been used to follow IVT amplifications on bovine oocytes and early embryos (Robert & Sirard, personal communication).

The possible differences between the sequences affected by amplification defaults were however rarely explored. Van Haaften observed that the reporters that disappeared after IVT amplification (20% of them) had a GC content of about 54% and displayed more hairpins of longer sizes than the other reporters (80%). A higher GC content has also been observed in deviating genes after PCR amplification with the SMART protocol [21]. The authors correlated this feature to the temperature of the enzymatic reaction (68 to 72°C for the Taq Polymerase) and to the GC content of their plant genome. This was surprising to us since GC rich fragments are often difficult PCR templates, requiring sometimes DMSO or betaine addition. In our study, we could not assign the distortions from Panel 1 and Panel 2 to IVT or PCR defaults since, without a standard, it was impossible to distinguish IVT over-expression from PCR under-expression and *vice versa*. It was clear however that these 2 gene subsets did not overlap: different molecular features, different signal intensities and different gene ID. EST from Panel 2 displayed reduced sizes, were more frequently located in the 3'end of the cDNAs and displayed a lower GC content than those from Panel 1. They also contained more hairpins (60% versus 37%) and A stretches (10% versus 5%) than those from Panel 1 but displayed similar contents of promoter-like sequences. Since EST corresponding to true differential expressions identified by PCR targets were frequently located at the 3'end of the referenced cDNAs and displayed reduced sizes (as compared to IVT specific ones), one would suggest that deviating genes from Panel 2 could display a PCR signature.

## Conclusion

From this work, it was not really possible to favour PCR over IVT amplification or *vice versa*. Both generated distortions and revealed true differential expressions between embryonic stages (minor intersect between differential patterns and biases), so that one would rather advise (i) using only one protocol to keep amplification factors and biases equal (ii) monitoring the amplification process as offered now through Real-Time PCR and (iii) searching for protocol specific expression differences or gene-protocol interactions before any differential analysis on a new dataset or a new array. Obviously, the choice between those protocols is also a question of total RNA input, time, cost and available arrays since amplified targets enriched in 3'end fragments will not hybridise to SSH fragments or 5'positioned oligos. Last but not least, knowing that Taq Polymerases make more mistakes than RNA polymerases do, IVT may be favoured over PCR to hybridise highly discriminating oligo-arrays or arrays from other species.

## Methods

### Bovine tissues

Estrus synchronized heifers of the Charolais breed were inseminated (day 0) and day 12 to day 17 blastocysts were collected by non surgical flushing in warm PBS. Ovoid blastocysts (1–12 mm) came from collects at 12 dpi (day post insemination) whereas tubular and early filamentous stages (50–60 mm and 140–160 mm) were obtained at 14 to 15 and 16 to 17 dpi, respectively. Brain and ovaries were collected on Day-50 pregnant cows. To take adult somatic tissues, animals were humanly put down in the accredited experimental slaughterhouse of INRA under the supervision of veterinary services.

### RNA extraction

Total RNA from ovoid (n = 4), tubular (n = 4) and filamentous (n = 4) embryos was extracted with RNA-Plus™ (QBioGene). RNA quality was first verified by intact ribosomal bands on a 1% agarose gel (28S and 18S) and A260/280 absorbance ratios. Total RNA from brain and ovary was isolated in the same way. RNA quality was also verified by intact ribosomal bands on a 1% agarose gel (28S and 18S) and A260/280 absorbance ratios. A spiking mRNA was then added to brain and ovary as 1% of the estimated polyA+ amount to test whether highly expressed genes can be biased through amplification. This CG03 mRNA from A. thaliana was in vitro synthesized (with a T7 Megascript kit, Ambion) from the c554 containing plasmid, given to us by H. Hofte (LBC, INRA Versailles, France). Brain and ovary polyA+ RNA were further extracted using a Dynabeads mRNA purification kit (Dynal).

### RNA amplification

Amplified RNA from each sample was synthesized with the MessageAmp™ aRNA Kit (Ambion) according to the manufacturer instructions. Briefly, 1 μg of total RNA was incubated with 500 ng of an anchored T7-(dT) primer in 12 μl (water) at 70°C for 10 min. The 1rst cDNA strand was synthesized by the addition of 2 μl first-strand buffer, 1 μl RNAse inhibitor, 4 μl dNTP mix and 1 μl reverse transcriptase mix and incubation at 42°C for 2 h. Second-strand synthesis was performed by the addition of 63 μl DEPC-treated water, 10 μl second-strand buffer, 4 μl dNTP mix, 2 μl DNA polymerase, 1 μl RNAse H- and incubation at 16°C for 2 h. DNA was extracted with phenol:chloroform:isoamyl alcohol and precipitated in ethanol with 20 μg glycogen (Ambion). In vitro transcription was carried out at 37°C for 10 h in a 20 μl reaction volume. 1 μl DNAse was added and incubated at 37°C for 30 min. RNA was purified on Mini Quick Spin RNA columns (Roche Diagnostic) and its quality verified on RNA 6000 lab-chips (BioAnalyser 2100; Agilent Technologies).

### RNA target labelling

aRNA was retro-transcribed and directly labelled with [α-^33^P]dATP as described for polyA+RNA [42]. 500 ng of aRNA was mixed with 500 ng of random hexamers in a volume of 25 μl. The mixture was incubated at 70°C for 10 min and chilled on ice. cDNA was synthesised by the addition of 5 μl 10× PCR buffer, 5 μl 25 mM MgCl_2_, 5 μl 0,1 mM DTT, 2,5 μl 10 mM mix dGTP, dCTP and dTTP, 2,5 μl water, 50 μCi [α-^33^P]dATP and 200 U Superscript II (Invitrogen) at 42°C for 50 min. The RNA template was removed by the addition of 1 μl RNAse H- and incubation at 37°C for 20 min.

### Global RT-PCR amplification

Amplified cDNA was prepared as described [24] with few modifications. Briefly, 1 μg total RNA was incubated with 1 μl 10 μM oligo(dT), 1 μl 10 mM dNTPs, 1 μl 10% NP40, 1 μl 20 mM DTT, 2 μl first-strand buffer 5×, 1 μl RNAse inhibitor (Ambion) at 65°C for 2 min, at room temperature for 3 min and cooled on ice. cDNA was synthesised by the addition of 200 U Superscript II (Invitrogen) and 2 U AMV (Gibco BRL) and incubation at 42°C for 30 min. First-strand cDNA were poly(dG)-tailed by incubation with 1 μl 20 mM dGTP, 4 μl TdT buffer 5×, 2,5 μl water, 2,5 μl TdT enzyme (Promega) at 37°C for 1 h. The first PCR was performed in a volume of 50 μl using 1/10 of the RT and the second PCR was performed on 1/4 of the first PCR. Samples were incubated at 94°C for 10 min before the two rounds of PCR cycles (12 cycles each; 94°C for 2 min, 63°C for 50 sec and 72°C for 6 min). PCR products were then purified using Qiaquick PCR purification (Qiagen) and their quality verified on DNA 7500 lab-chips (BioAnalyser 2100; Agilent Technologies).

### cDNA target labelling

PCR-amplified cDNA was labelled with [α-^33^P]dATP using random hexamers and Klenow included in Atlas SMART Probe Amplification kit (Clontech). 500 ng of amplified cDNA was mixed with 500 ng random hexamers in a volume of 34 μl. The mixture was incubated at 98°C for 8 min and at 50°C for 3 min. After addition of 5 μl 10× buffer, 5 μl dNTPs for ATP label, 5 μl [α-^33^P]dATP and 1 μl Klenow, the reaction mixture was incubated at 50°C for 30 min and stopped with 2 μl 0,5 M EDTA. Labelled targets were then purified on Sephadex columns (G-50).

### Quantitative Real time PCR

Real-time PCR was carried out in a final volume of 30 μl with 1 μl of diluted reverse transcriptions (1/100; 1/1000) in a 1× SYBR green Master Mix (Applied Biosystems) with 0.3 μM of gene-specific primers. Reactions were run on ABI Prism 7000 HT (Applied Biosystems). The presence of a specific and unique PCR product was checked by ABI Prism melting curves. The relative quantification of the initial amount of target was extrapolated from the appropriate standard curve, which was generated simultaneously while using serial dilutions of the corresponding PCR product. IFN-tau and Gapdh primers were as published [43,44] but Cox2 primers were a kind gift from G. Charpigny. Their sequences (unpublished so far) will be available upon request gilles.charpigny@jouy.inra.fr.

### Slot-Blot

125 ng of polyA+RNA, aRNA or cDNA were spotted and cross-linked to HybondN+ membranes (Amersham) at 80°C for 2 h. DNA probes encoding IFN-tau, CG03, EF1α, RPL23a or Cytochrome oxidase III were [α-^32^P]dCTP-labelled using the Ready-Prime kit (Amersham). Apart from CG03, those DNA probes originate from the array. Hybridisations were conducted at 65°C for 16 h and washes performed once in 2 × SSC, 0,1% SDS at 65°C for 30 min and twice in 0,1 × SSC, 0,1% SDS at 65°C for 10 min. Slot blots were then exposed to phosphor-imaging for 24 hours and signal intensities quantified with the ImageQuant 3.3 software (Molecular Dynamics).

### Array description

The bovine embryonic array used here originates from a bovine cDNA library established at the ovoid stage, starting from 1.6 μg of RNA and using the Cap Finder cDNA kit from Clontech as described in Degrelle et al. [26]. Briefly, cDNA inserts from the arrayed library were amplified by PCR using the flanking primers from the Cap Finder kit and selected for spotting after a short run on a 2.5% agarose gel. 1855 probes were then spotted and fixed (UV light, 1 min, 1200 J, twice) onto nylon N+ membranes (8 cm × 12 cm, Amersham Biosciences) with a 5 × 5 pattern (BioRobotics). This was achieved with the kind help of C. Matingou and G. Piétu at the Genexpress Laboratory headed by C. Auffray (CNRS FRE 2571, Villejuif, France). The library has been called "bcai" and indexed in TGI and NCBI database as "#FJB" and "15979", respectively [45,46] and the array published as "INRA-BDR Bovine D14 Embryo 1K" (GPL6284) in NCBI Gene Expression Omnibus database [47]. Bacterial clones are available upon request at the CRB GADIE (INRA Jouy en Josas, France [48])

### Array hybridization, image acquisition and quantification

Each target was hybridized to 4 array replicates using ExpressHyb™ Hybridization Solution (Clontech) at 68°C overnight. Arrays were washed four times in 2 × SSC, 1% SDS and once in 0.1 × SSC, 0.5% SDS at 68°C for 30 min each. They were then exposed to phosphor-screens for 7 days. The hybridization signals were quantified with the Imagene 3.1 software from BioDiscovery (Proteigene) on the PICT plateform (INRA Jouy en Josas, France). These raw datasets are accessible in NCBI Gene Expression Omnibus database (experimental series "GSE9929" [47]). Internal controls within the array corresponded to 65 probes and either positive or negative controls were as expected in all the hybridizations. A signal was considered "valid" when the Imagene software did not flag it (flag = 0) and when the same signal was observed on 2 thirds of the arrays, namely: 8 out of 12 for the somatic targets and 5 out of 8 for the embryonic ones.

### Gene expression analyses

All the plots (scatter plots, histograms) were performed on R environment [49].

#### Gene expression differences between protocols

These analyses were performed either on the whole array (1855 inserts plus 65 controls = 1920 probes) or on the biological core of the array also called 1 K array (1097 informative sequences submitted to the EBI – 110 mitochondrial sequences = 987 probes). With 2 protocols, 5 tissues, 2 to 3 target replicates per protocol and 4 array replicates per target (as indicated in the experimental design, Fig. 1), these analyses involved 184 320 (1920*2*2*3*4+1920*2*3*2*4) and 94 752 (987*2*2*3*4+987*2*3*2*4) pieces of data, respectively. Statistical and clustering analyses were performed using TIGR MeV 3.0 (MultiExperiment Viewer software [50]). Before calculations, the data were log2 transformed and standardised within each protocol. Differences between PCR and IVT methods were assessed by a Student's t-test assuming an unequal variance (Welch approximation). The adjusted Bonferroni correction was considered at P < 0.05. An unsupervised hierarchical clustering, based on Euclidean distance and complete linkage, was performed on the significant gene expression differences between the 2 methods.

#### Gene expression differences between embryonic stages

These analyses were performed on the biological core of the array (987 probes). With 2 protocols, 3 embryonic stages, 2 target replicates per protocol and 4 array replicates per target (as indicated in the design), these analyses involved 47 376 (987*3*2*2*4) pieces of data. To identify gene expression differences between stages, we used a set of SAS macros called AnovArray and performed an analysis of variance considering a homogeneous variance for all the genes (HOM option) and a multiple testing (False Discovery Rate) at the threshold 5% (details in [26,51]). AnovArray has been originally conceived to analyse these datasets.

### Bioinformatics

Biological processes were analysed through Gene Ontology annotations [52] considering the Indentation 1. EST size, GC content, EST position according to the referenced mRNA of the Bos taurus gene index [45] were performed using Perl scripts and box plot function from the R environment [49]. Presence of hairpins, dA stretches and sequences similar to RNA polymerase promoters was evaluated using the *palindrome *and *fuzznuc *programs of the Emboss package [53].

## Authors' contributions

SAD provided and analyzed the data. CHA, KPK, SR developed AnovArray. HC contributed to bioinformatic analyses. FP took part to the 1 K array construction. JPR helped conceiving the study. IH, SAD designed the study and wrote the manuscript. All authors read and approved the final manuscript.

## Supplementary Material

Additional file 1 **Optimisation of each amplification procedure**. Southern (A) and Northern (B) blots performed on cDNA (A) and aRNA (B) after increasing PCR cycle numbers or increasing in vitro transcription times were hybridised with a ^32 ^P-labelled DNA probe encoding the exogenous CG03 transcript. A band of the expected size (1 kb) was observed on southern blots after 9, 12 and 15 cycles for the 1^rst ^and 2^nd ^rounds of PCR amplifications (A). The negative controls including RT- and mock did not give any signal. A band of the expected size was also observed on Northern blots after 8, 10 or 12 h of in vitro transcription (B). Its intensity increased with the increasing transcription time. Only brain data are illustrated here, but similar results were obtained with ovary and embryos. Click here for file

Additional file 2 **Characteristics of the amplified targets from brain and ovary**. aRNA and cDNA targets were analysed on RNA 6000 lab-chips and DNA 7500 lab-chips, respectively (BioAnalyser 2100; Agilent Technologies). These populations of amplified molecules displayed slightly different profiles of size distribution depending on the protocol (A, B) or the tissue (C). Each target replicate (1 to 3) has been amplified independently from the same pool of total RNA. The molecular ladders are represented in nucleotides (nt) on the x axis. Click here for file

Additional file 3 **List of the 109 EST from Panel 1**. Name of the EST from the 1 K array (or core array), GenBank accession numbers (CR), identifiers in TIGR gene index (TC) and Unigene index (Bt.) as well as short names (Gene ID) are provided here. Click here for file

Additional file 4 **List of the 45 EST from Panel 2**. Name of the EST from the 1 K array (or core array), GenBank accession numbers (CR), identifiers in the TIGR gene index (TC) and the Unigene index (Bt.) as well as short names (Gene ID) are provided here. Click here for file
