# Supplementary figures and images for: Amplification biases: possible differences among deviating gene expressions
Source: BMC Genomics. 2008 Jan 28;9:46. doi: 10.1186/1471-2164-9-46 (PMC2257942; doi:10.1186/1471-2164-9-46)

**A****cDNA**

---

RT-    9x    12x    15x    mock

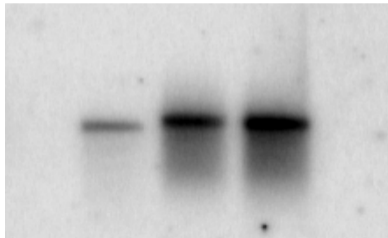**-1 Kb****B****aRNA**

---

8h    10h    12h

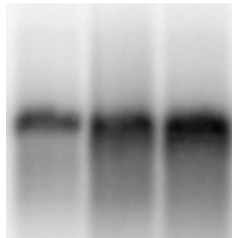**-1 Kb**

Supplement: Additional file 1 — Optimisation of each amplification procedure. Southern (A) and Northern (B) blots performed on cDNA (A) and aRNA (B) after increasing PCR cycle numbers or increasing in vitro transcription times were hybridised with a 32 P-labelled DNA probe encoding the exogenous CG03 transcript. A band of the expected size (1 kb) was observed on southern blots after 9, 12 and 15 cycles for the 1rst and 2nd rounds of PCR amplifications (A). The negative controls including RT- and mock did not give any signal. A band of the expected size was also observed on Northern blots after 8, 10 or 12 h of in vitro transcription (B). Its intensity increased with the increasing transcription time. Only brain data are illustrated here, but similar results were obtained with ovary and embryos. [file 1471-2164-9-46-S1.PDF]

**A**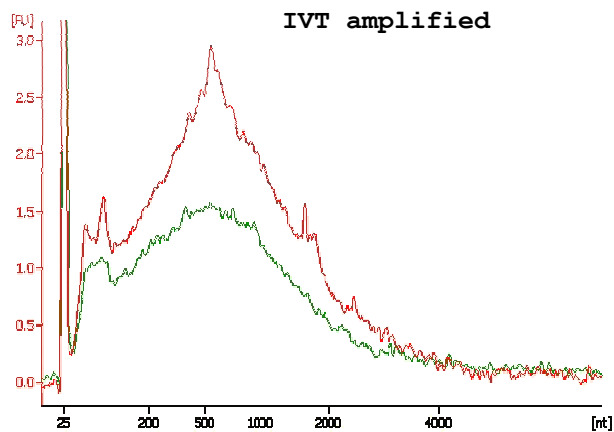**B**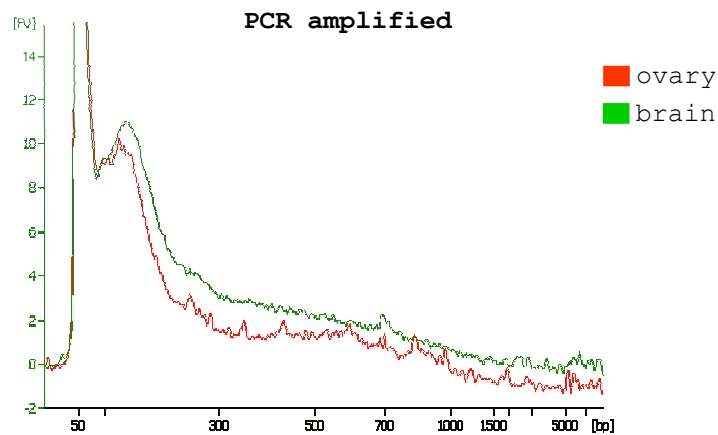**C**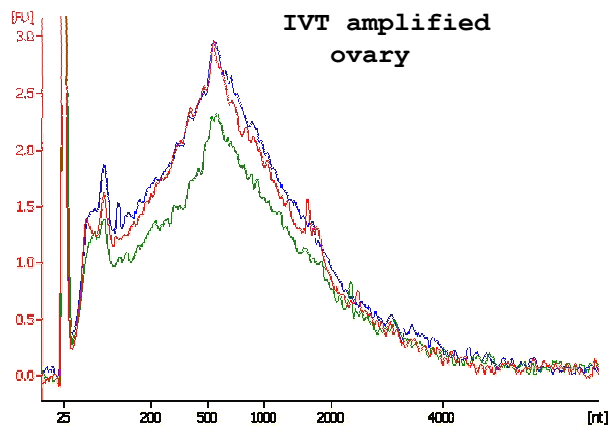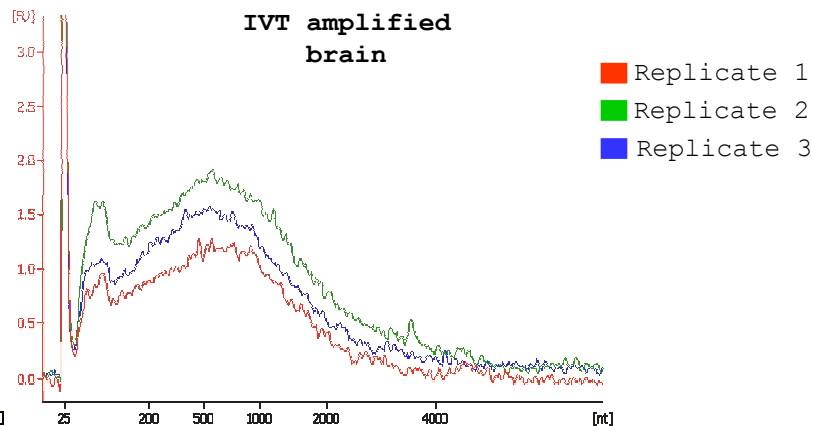

Supplement: Additional file 2 — Characteristics of the amplified targets from brain and ovary. aRNA and cDNA targets were analysed on RNA 6000 lab-chips and DNA 7500 lab-chips, respectively (BioAnalyser 2100; Agilent Technologies). These populations of amplified molecules displayed slightly different profiles of size distribution depending on the protocol (A, B) or the tissue (C). Each target replicate (1 to 3) has been amplified independently from the same pool of total RNA. The molecular ladders are represented in nucleotides (nt) on the x axis. [file 1471-2164-9-46-S2.PDF]
