# Supplementary material for: Amplification biases: possible differences among deviating gene expressions
Source: BMC Genomics. 2008 Jan 28;9:46. doi: 10.1186/1471-2164-9-46 (PMC2257942; doi:10.1186/1471-2164-9-46)
Supplement: Additional file 3 — List of the 109 EST from Panel 1. Name of the EST from the 1 K array (or core array), GenBank accession numbers (CR), identifiers in TIGR gene index (TC) and Unigene index (Bt.) as well as short names (Gene ID) are provided here. [file 1471-2164-9-46-S3.PDF]

109 EST from Panel 1

1K array EST	GenBank	TIGR	Bt.Unigene	Gene ID
bcai0001a.b.07_5.1	CR451538	TC289253	Bt.14186	ACTB_TRIVU
bcai0001a.c.02_5.1	CR451518	TC260206	Bt.14838	COX7R_HUMAN
bcai0001a.c.03_5.1	CR451496	TC262633	Bt.5051	UBIQ_XENLA
bcai0001a.c.06_5.1	CR451476	TC267064	Bt.27116	Bt.27116
bcai0001a.e.04_5.1	CR455233	TC289292	Bt.2686	RS16_RAT
bcai0001a.e.09_5.1	CR455212	TC260184	Bt.3091	RL23_RAT
bcai0001a.e.11_5.1	CR455190	TC262660	Bt.4431	ATPB_BOVIN
bcai0001a.h.11_5.1	CR450879	TC276636	Bt.5052	RAN_RAT
bcai0001a.h.12_5.1	CR450880	TC276461	Bt.7194	ATPA1_BOVIN
bcai0002a.b.10_5.1	CR453169	TC277067	Bt.886	PSA5_MOUSE
bcai0002a.c.01_5.1	CR451190	TC262472	Bt.10306	AY526085
bcai0002a.d.06_5.1	CR455039	TC275216	Bt.15534	TBAK_HUMAN
bcai0002a.f.12_5.1	CR455311	TC262605	Bt.23381	RL10A_RAT
bcai0002a.g.03_5.1	CR455326	TC262217	Bt.3573	EF1A1_RABIT
bcai0002a.g.11_5.1	CR455315	TC262217	Bt.3573	EF1A1_RABIT
bcai0002a.h.04_5.1	CR455319	.	Bt.22533	ALDOA_RAT
bcai0003a.f.04_5.1	CR451398	TC274725	Bt.30085	ROD_HUMAN
bcai0003a.f.05_5.1	CR451399	TC262487	Bt.5466	RS4X_RAT
bcai0003a.f.06_5.1	CR451400	TC261685	Bt.9814	RL36A_RAT
bcai0003a.f.09_5.1	CR451403	TC293505	Bt.33879	Bt.33879
bcai0003a.g.02_5.1	CR451365	TC289253	Bt.14186	ACTB_TRIVU
bcai0004a.b.01_5.1	CR451382	TC276662	Bt.2515	H2AZ_ONCMY
bcai0004a.b.10_5.1	CR451146	TC260284	Bt.8015	RS24_XENLA
bcai0004a.c.12_5.1	CR451155	TC263331	Bt.6473	PSA7_MOUSE
bcai0004a.e.08_5.1	CR451128	TC262595	Bt.4967	RS3A_HUMAN
bcai0005a.a.05_5.1	CR451078	TC289253	Bt.14186	ACTB_TRIVU
bcai0005a.a.11_5.1	CR451081	TC275739	.	Bt.31065
bcai0005a.b.06_5.1	CR451086	TC275281	Bt.23254	1433T_HUMAN
bcai0005a.b.08_5.1	CR451088	TC289253	Bt.14186	ACTB_TRIVU
bcai0005a.c.03_5.1	CR451090	TC289918	Bt.676	H2AV_CHICK
bcai0005a.c.06_5.1	CR451091	.	.	YO11_MOUSE
bcai0005a.c.09_5.1	CR451094	TC266174	Bt.9825	RM35_MOUSE
bcai0005a.c.10_5.1	CR451095	.	.	05c10
bcai0005a.h.12_5.1	CR455054	TC262217	Bt.3573	EF1A1_RABIT
bcai0006a.b.08_5.1	CR455025	TC290591	Bt.11936	DUT_HUMAN
bcai0006a.c.09_5.1	CR455033	TC262217	Bt.3573	EF1A1_RABIT
bcai0006a.d.04_5.1	CR455000	TC289278	Bt.4967	RS3A_HUMAN
bcai0006a.d.06_5.1	CR942253	TC289253	Bt.14186	ACTB_TRIVU
bcai0006a.d.07_5.1	CR455002	TC289253	Bt.14186	ACTB_TRIVU
bcai0006a.g.03_5.1	CR454980	TC262450	Bt.5334	RSSA_BOVIN
bcai0006a.g.10_5.1	CR454985	TC276111	.	Bt.29283
bcai0007a.a.04_5.1	CR454996	TC262595	Bt.4967	RS3A_HUMAN
bcai0007a.a.11_5.1	CR454961	TC262740	Bt.3779	RS15A_RAT
bcai0007a.b.06_5.1	CR454966	TC276729	Bt.742	HMG1_RAT
bcai0007a.b.12_5.1	CR454970	TC260184	Bt.3091	RL23_RAT
bcai0007a.e.04_5.1	CR451251	TC290573	Bt.5397	SPC18_HUMAN
bcai0007a.f.11_5.1	CR451222	TC261082	Bt.742	HMG1_RAT
bcai0007a.g.02_5.1	CR451224	TC289846	Bt.4880	MPCP_BOVIN
bcai0007a.g.11_5.1	CR451229	TC262000	Bt.5471	IF4G2_RABIT
bcai0007a.h.03_5.1	CR451192	TC262591	Bt.12309	HS7C_SAGOE
bcai0008a.b.01_5.1	CR451207	TC262217	Bt.3573	EF1A1_RABIT
bcai0008a.b.03_5.1	CR451208	TC289253	Bt.14186	ACTB_TRIVU
bcai0008a.c.08_5.1	CR451180	TC276703	Bt.7785	EGD2_YEAST
bcai0008a.c.09_5.1	CR451181	TC261639	Bt.23343	RL27A_RAT

1K array EST	GenBank	TIGR	Bt.Unigene	Gene ID
bcai0008a.f.04_5.1	CR451159	TC274824	Bt.4620	PCB1_RABIT
bcai0008a.f.07_5.1	CR451161	TC289253	Bt.14186	ACTB_TRIVU
bcai0008a.f.11_5.1	CR451165	TC261497	Bt.16991	EF1B_HUMAN
bcai0008a.g.08_5.1	CR451168	TC289361	Bt.4035	1433E_SHEEP
bcai0008a.g.09_5.1	CR453149	TC261610	.	Bt.3212
bcai0008a.h.03_5.1	CR453155	TC289073	Bt.3036	RL11_RAT
bcai0009a.a.01_5.1	CR453162	TC274823	Bt.5245	RS2_RAT
bcai0009a.c.08_5.1	CR453143	TC277067	Bt.886	PSA5_MOUSE
bcai0009a.e.12_5.1	CR453121	TC261685	Bt.9814	RL36A_RAT
bcai0010a.a.11_5.1	CR450945	TC278339	Bt.404	ATP5J_BOVIN
bcai0010a.b.10_5.1	CR450952	TC278339	Bt.404	ATP5J_BOVIN
bcai0010a.d.07_5.1	CR450927	.	Bt.7648	RS19_HUMAN
bcai0010a.e.11_5.1	CR450935	TC289253	Bt.14186	ACTB_TRIVU
bcai0010a.f.02_5.1	CR450937	TC275190	Bt.5245	RS2_RAT
bcai0011a.b.08_5.1	CR450893	TC274828	Bt.14228	PRL_BOVIN
bcai0011a.d.02_5.1	CR450867	TC260235	Bt.8094	G25L2_HUMAN
bcai0012a.b.03_5.1	CR450999	TC289253	Bt.14186	ACTB_TRIVU
bcai0012a.b.11_5.1	CR451007	TC260232	Bt.3867	AT5F1_BOVIN
bcai0012a.c.10_5.1	CR451013	TC262591	Bt.12309	HS7C_SAGOE
bcai0012a.f.06_5.1	CR450991	TC279177	Bt.6300	SC61G_MOUSE
bcai0012a.f.12_5.1	CR450995	TC267669	Bt.3583	EZRI_BOVIN
bcai0013a.a.06_5.1	CR451421	TC289240	Bt.22818	COX3_MOUSE
bcai0013a.b.01_5.1	CR451428	TC276919	Bt.2847	MYL6_RAT
bcai0013a.f.02_5.1	CR451458	TC263098	Bt.1129	CISY_HUMAN
bcai0013a.f.03_5.1	CR942250	TC276418	Bt.3616	RL3_BOVIN
bcai0013a.h.03_5.1	CR455173	TC263112	Bt.2816	ATP5L_BOVIN
bcai0013a.h.12_5.1	CR455180	TC289253	Bt.14186	ACTB_TRIVU
bcai0014a.a.04_5.1	CR455181	TC289253	Bt.14186	ACTB_TRIVU
bcai0014a.e.03_5.1	CR455213	TC289306	Bt.7863	RS3_HUMAN
bcai0014a.e.08_5.1	CR455216	TC262595	Bt.4967	RS3A_HUMAN
bcai0014a.f.12_5.1	CR455224	TC289240	Bt.39132	AY526085
bcai0014a.g.08_5.1	CR455231	TC262633	Bt.5051	UBIQ_XENLA
bcai0014a.h.09_5.1	CR455240	TC289253	Bt.14186	ACTB_TRIVU
bcai0015a.a.10_5.1	CR455247	TC260183	Bt.15931	SPRC_HUMAN
bcai0015a.d.03_5.1	CR455332	.	.	UGG2_HUMAN
bcai0015a.e.07_5.1	CR455344	TC289282	Bt.23291	CALM_XENLA
bcai0016a.a.10_5.1	CR455378	TC261663	Bt.5542	NP1L1_HUMAN
bcai0016a.e.10_5.1	CR455412	TC289253	Bt.14186	ACTB_TRIVU
bcai0016a.f.06_5.1	CR455418	TC289073	Bt.3036	RL11_RAT
bcai0016a.h.04_5.1	CR451463	TC289429	Bt.5054	RL24_RAT
bcai0017a.c.02_5.1	CR451491	TC263531	Bt.9540	OM07_HUMAN
bcai0017a.d.09_5.1	CR451502	TC261642	Bt.32873	VPRT_JSRV
bcai0017a.e.04_5.1	CR451507	TC263531	Bt.9540	OM07_HUMAN
bcai0017a.g.01_5.1	CR451523	TC289253	Bt.14186	ACTB_TRIVU
bcai0017a.g.11_5.1	CR451527	TC289253	Bt.14186	ACTB_TRIVU
bcai0018a.b.08_5.1	CR451548	TC274368	Bt.12659	PWP1_HUMAN
bcai0018a.e.02_5.1	CR451263	TC274823	Bt.5245	RS2_RAT
bcai0018a.e.03_5.1	CR451264	TC262633	Bt.5051	UBIQ_XENLA
bcai0018a.h.05_5.1	CR450966	TC261605	Bt.5542	NP1L1_HUMAN
bcai0019a.a.05_5.1	CR450972	.	Bt.18007	Bt.18007
bcai0019a.f.07_5.1	CR451353	TC289253	Bt.14186	ACTB_TRIVU
bcai0019a.g.08_5.1	CR451321	TC262065	Bt.22737	RL6_HUMAN
bcai0019a.h.10_5.1	CR451332	TC274824	Bt.4620	PCB1_RABIT
bcai0020a.a.06_5.1	CR451334	TC277210	Bt.14010	LB4D_PIG
bcai0020a.b.09_5.1	CR451301	TC276461	Bt.7194	ATPA1_BOVIN
