# Supplementary material for: Amplification biases: possible differences among deviating gene expressions
Source: BMC Genomics. 2008 Jan 28;9:46. doi: 10.1186/1471-2164-9-46 (PMC2257942; doi:10.1186/1471-2164-9-46)
Supplement: Additional file 4 — List of the 45 EST from Panel 2. Name of the EST from the 1 K array (or core array), GenBank accession numbers (CR), identifiers in the TIGR gene index (TC) and the Unigene index (Bt.) as well as short names (Gene ID) are provided here. [file 1471-2164-9-46-S4.PDF]

45 EST from Panel 2

1K array EST	GenBank	TIGR	Bt.Unigene	Gene ID
bcai0001a.c.04_5.1	CR451497	TC264955	.	ARF3_RAT
bcai0001a.d.01_5.1	CR455381	.	Bt.14228	AB098876
bcai0001a.e.01_5.1	CR455253	TC274814	Bt.24696	Bt.24696
bcai0001a.g.05_5.1	CR450976	TC262815	Bt.9548	RL39_ICTPU
bcai0002a.b.02_5.1	CR453126	TC263978	Bt.15779	KRAC_DICDI
bcai0002a.b.06_5.1	CR453147	TC292763	Bt.22027	Bt.22027
bcai0002a.c.03_5.1	CR451211	TC294446	.	Bt.25225
bcai0002a.c.09_5.1	CR451254	TC279709	Bt.28502	CT129_HUMAN
bcai0002a.d.02_5.1	CR455018	TC292235	Bt.15307	Bt.15307
bcai0002a.e.01_5.1	CR451077	TC261729	.	TF1B_HUMAN
bcai0002a.g.10_5.1	CR455314	TC264574	Bt.27027	RT15_HUMAN
bcai0003a.e.01_5.1	CR451392	TC261729	.	TF1B_HUMAN
bcai0003a.f.08_5.1	CR451402	TC293505	.	Bt.33879
bcai0003a.g.06_5.1	CR451367	TC288058	.	AT8A1_BOVIN
bcai0003a.h.07_5.1	CR451372	TC263422	Bt.4339	CT027_HUMAN
bcai0004a.g.06_5.1	CR451104	TC295528	Bt.39526	Hs.54037
bcai0005a.d.02_5.1	CR451058	TC294102	Bt.14278	BCLF1_HUMAN
bcai0005a.f.06_5.1	CR451073	.	Bt.41670	DHBX_ANAPL
bcai0005a.h.07_5.1	CR455051	TC262815	Bt.9548	RL39_ICTPU
bcai0006a.c.02_5.1	CR455030	TC290668	Bt.3254	PAC2_YEAST
bcai0007a.c.08_5.1	CR451235	.	.	EFB2_MOUSE
bcai0007a.d.04_5.1	CR451241	TC262735	Bt.26365	TKDP1_SHEEP
bcai0007a.h.10_5.1	CR451196	.	.	Bt.33716
bcai0008a.e.04_5.1	CR942251	TC278354	Bt.4702	Bt.4702
bcai0008a.g.10_5.1	CR453150	TC288058	.	AT8A1_BOVIN
bcai0008a.h.01_5.1	CR453153	TC260381	Bt.4942	SERF2_MOUSE
bcai0009a.a.07_5.1	CR453166	TC267531	Bt.41668	140U_DROME
bcai0009a.g.06_5.1	CR453088	TC264113	Bt.25106	YHH2_SCHPO
bcai0009a.h.06_5.1	CR453096	TC264358	Bt.3541	Bt.3541
bcai0010a.h.04_5.1	CR450912	.	.	Bt.19598
bcai0011a.b.05_5.1	CR450890	.	.	Bt.33709
bcai0012a.b.10_5.1	CR451006	TC288058	.	AT8A1_BOVIN
bcai0012a.e.05_5.1	CR450983	TC276987	Bt.11242	BR44L_HUMAN
bcai0012a.f.04_5.1	CR450989	.	.	Bt.16383
bcai0013a.e.08_5.1	CR451454	TC260263	.	Bt.33708
bcai0013a.h.10_5.1	CR455178	TC289262	Bt.5535	1433B_HUMAN
bcai0016a.b.10_5.1	CR455386	TC280482	.	CEP27_HUMAN
bcai0016a.c.04_5.1	CR455388	TC290624	Bt.4007	HAX1_HUMAN
bcai0016a.d.02_5.1	CR455397	.	Bt.37972	Hs.5737
bcai0016a.g.05_5.1	CR455426	.	Bt.28078	Bt.6457
bcai0017a.a.05_5.1	CR451472	TC261339	Bt.4798	ACOD_BOVIN
bcai0017a.b.06_5.1	CR451486	TC266572	Bt.22385	MGN_XENLA
bcai0018a.b.06_5.1	CR451546	TC289409	Bt.435	TIM2_BOVIN
bcai0020a.g.09_5.1	CR451288	TC281068	Bt.12850	GLMN_HUMAN
bcai0020a.h.09_5.1	CR451295	TC291677	Bt.20133	RBP6_HUMAN
